# Sequencing and De Novo Assembly of the *Toxicodendron radicans* (Poison Ivy) Transcriptome

**DOI:** 10.3390/genes8110317

**Published:** 2017-11-10

**Authors:** Alexandra J. Weisberg, Gunjune Kim, James H. Westwood, John G. Jelesko

**Affiliations:** 1 Department of Botany and Plant Pathology, Oregon State University, Corvallis, OR 97330, USA; weisbeal@oregonstate.edu; 2 Department of Plant Pathology, Physiology, and Weed Science, Virginia Tech, Blacksburg, VA 24061, USA; gunjunekim@gmail.com (G.K.); westwood@vt.edu (J.H.W.)

**Keywords:** *Toxicodendron radicans*, poison ivy, urushiol, contact dermatitis, skin rash, transcriptome, Anacardiaceae

## Abstract

Contact with poison ivy plants is widely dreaded because they produce a natural product called urushiol that is responsible for allergenic contact delayed-dermatitis symptoms lasting for weeks. For this reason, the catchphrase most associated with poison ivy is “leaves of three, let it be”, which serves the purpose of both identification and an appeal for avoidance. Ironically, despite this notoriety, there is a dearth of specific knowledge about nearly all other aspects of poison ivy physiology and ecology. As a means of gaining a more molecular-oriented understanding of poison ivy physiology and ecology, Next Generation DNA sequencing technology was used to develop poison ivy root and leaf RNA-seq transcriptome resources. De novo assembled transcriptomes were analyzed to generate a core set of high quality expressed transcripts present in poison ivy tissue. The predicted protein sequences were evaluated for similarity to SwissProt homologs and InterProScan domains, as well as assigned both GO terms and KEGG annotations. Over 23,000 simple sequence repeats were identified in the transcriptome, and corresponding oligo nucleotide primer pairs were designed. A pan-transcriptome analysis of existing Anacardiaceae transcriptomes revealed conserved and unique transcripts among these species.

## 1. Introduction

Poison ivy (*Toxicodendron radicans* (L.) Kuntze) is widely known for its ability to induce allergenic contact delayed-dermatitis (poison ivy rash) symptoms that can last for weeks on between 25–40 million people per year in North America [1,2,3,4]. The poison ivy alkylphenol natural product responsible for the allergenic dermatitis is called urushiol [5]. Urushiol is not a single compound but rather a collection of penta- and hepta-dec(en)yl-catechol congeners that can vary in degrees of unsaturation ranging from zero to three double bonds [6,7,8,9]. Urushiol is present in all poison ivy organs [10] and accumulates within resin ducts/canals [11]. Despite the notoriety of poison ivy as a human contact allergen, there are surprisingly few peer reviewed studies in which poison ivy ecology [12,13,14] or non-urushiol physiology [15,16] are the primary topic of the study.

Poison ivy shows a high degree of natural phenotypic variation across its natural range. Variation in leaf margin lobing and pubescence of both leaves and fruit that generally align with different North American geographical regions led William Gillis to assign subspecies to *T. radicans* ssp.: *barkleyi*, *divaricatum*, *eximium*, *negundo*, *pubens*, *radicans*, and *verrucosum* [12,17]. However, these subspecies designations can be complicated by apparent hybrid intergrade zones. Interestingly, two additional subspecies of poison ivy, *T. radicans* ssp. *orientale* and ssp. *hispidium*, are geographically disjoined in east Asia [12]. Poison ivy shows three distinct growth habits: shrub, ground creeping stolons, and climbing lianas. This flexibility in growth habit allows poison ivy to exploit a variety of habitats, including habitats that have undergone natural disruption. Poison ivy incidence has been shown to greatly increase after either hurricane-induced [18,19] or range fire-induced [12,20] habitat disturbance.

Poison ivy ecology, especially urushiol chemical ecology, is currently enigmatic. The familiarity of human allergenic dermatitis and urushiol accumulation in all poison ivy organs, suggests that urushiol is a potent chemical defense in natural habitats. While this may well prove to be the case, the presumed ecological targets of urushiol chemical defense have yet to be conclusively demonstrated. Wild and domesticated mammals (bear, deer, mice, rabbit, squirrel, and goats) eat poison ivy leaves or drupes [21,22,23]. Likewise, a variety of wild birds eat poison ivy drupes [13,21,24]. However, none of these fauna display demonstrable adverse effects from consuming these poison ivy organs. A number of insect species are reported to interact with poison ivy [25,26]; but whether urushiol acts as a chemical defense against these herbivorous insects has not been established.

Most aspects of poison ivy physiology are also poorly characterized. Poison ivy is a dioecious plant, exhibiting at least three years of establishment prior to flowering [12]. A urushiol biosynthetic pathway is proposed [27,28], but none of the suggested biosynthetic steps have been biochemically or genetically validated. The geographically distributed *T. radicans* subspecies across North America suggest that poison ivy is readily adapting to local environments [12]. Consistent with this notion, three geographically-isolated poison ivy accessions showed significant accession-level differences in a range of biometric traits in a common garden experiment in which light and nutrient conditions were manipulated [29]. In 2006, poison ivy was shown to respond to elevated atmospheric CO_2_ levels by growing faster, producing more biomass, and producing a urushiol congener composition that is significantly enriched in more unsaturated pentadecyltriene-catechol congeners [15,16] that are associated with an increased severity of allergenic dermatitis in humans [30]. These findings suggest that expected patterns of climate change will result in poison ivy that is both more weedy and more noxious.

Next Generation DNA sequencing technologies have the potential to broadly advance poison ivy research. In particular, Illumina sequencing can provide both deep sampling and de novo assembly of plant transcriptomes derived from total plant RNA. Such transcriptomes can serve as both the basis of gene discovery and the quantification of gene expression levels. Here, we report the first de novo RNA-seq transcriptomes from poison ivy leaves and roots, as well as a comparative analysis of the currently sequenced Anacardiaceae family transcriptomes.

## 2. Materials and Methods

### 2.1. Axenic Cultured Seedlings and Plant Tissue

*T. radicans* subsp. *radicans* drupes from the VARoaCo-1 liana were germinated and grown under sterile culture conditions as previously described [31]. A typical VARoaCo-1 seedling voucher herbarium specimen (#109165) was deposited in the Virginia Tech Massey Herbarium. Drupes were plated at 20 seeds per 150 mm × 15 mm Petri plate containing sterile 0.5× MS Basal Salts media pH 5.7 [32] and 0.3% *w/v* Phytagel (Sigma-Aldrich Co., St. Louis, MO, USA). After four days, drupes in closed Petri plates were examined under a Leica Zoom 2000 illuminated stereo microscope (20× magnification) for an absence of microbial growth. Visually determined axenic seedlings were transferred to individual Magenta boxes or Phytatray II boxes (Sigma-Aldrich Co.) containing 0.5× MS Basal Salts media with 0.3% *w/v* Phytagel and then placed in a Percival CU-36l4 growth chamber (Percival Scientific, Perry, IA, USA) at 28 °C and 16 h light.

### 2.2. RNA Purification and Sequencing

Total RNA was extracted from two independent replications of either true leaves or roots harvested from 10–12 combined axenic poison ivy plants using a phenol/chloroform/SDS extraction protocol [33]. A 0.2 M KOAc treatment was included to precipitate and remove polysaccharides prior to the precipitation of total nucleic acids. RNA was selectively purified using a 2 M LiCl_2_ precipitation step, followed by subsequent purification using a Qiagen RNeasy Mini Kit (Qiagen, Venlo, The Netherlands) until an A260/280 of at least 1.8 was reached. RNA samples were submitted to the Virginia Bioinformatics Institute, Blacksburg, VA, for analysis using a BioAnalyzer (Agilent Genomics, Santa Clara, CA, USA), library preparation, and sequencing on an Illumina HiSeq 2000 (Illumina, San Diego, CA). Each of the four samples was selected for poly-A sequences and run on individual lanes of the HiSeq 2000.

### 2.3. De Novo Assembly of Poison Ivy Transcripts

The quality of the sequenced paired-end reads produced in each sample was observed using FastQC 0.10.1 [34] before and after trimming using Trimmomatic 0.3 [35] (Table 1). Paired-end reads were trimmed to remove Illumina sequencing adaptors, as well as portions of poor quality reads using a sliding window analysis (Trimmomatic settings: “LEADING:3 TRAILING:3 SLIDING-WINDOW:4:15 MINLEN:36”). The combined paired reads from all four samples were *de novo* assembled into an initial transcriptome using Trinity RNA Seq release 14 August 2013 [36]. Reads were then aligned to transcripts using Bowtie 2.1.0 [37] to assess overall assembly quality and analyze individual transcripts. Sequencing contaminants and overall assembly quality were quantified using the blobtools package [38] employing BLASTX search results (NCBI nr database, *e*-value 10) to assign transcripts to taxa. A subsequent reference de novo assembly and analysis of the three high quality samples (without the contaminated Root A sample) was performed using Trinity version 2.4.0. Transcriptome completeness was assessed using BUSCO 3.0.1 [39] with the embryophyta_odb9 dataset.

Assembled transcripts were annotated using Trinotate v 3.0.1 [40], as well as several other programs, including HMMER [41] to predict PFAM domains [42], SignalP [43], tmHMM [44], and RNAmmer [45]. TransDecoder v. 3.0.1 [40] was used to predict open reading frames (ORFs) and predicted translated protein sequences from assembled transcripts. Each transcript was used as a query in a BLASTX search (default parameters, *e*-value 10) against the SwissProt and NCBI nr databases; predicted protein sequences were also used as queries in BLASTP searches. All annotations were then combined using Trinotate. GO terms and KEGG classifications were assigned to transcripts utilizing Trinotate and the SwissProt BLASTX hits.

The Trinity pipeline suggested protocol and helper scripts were used to estimate transcript abundance and compare expression across samples. Paired-end reads from each of the three high quality samples were aligned to the assembled transcripts using Bowtie2 v.2.1.0 [37] with the default parameters, and converted to sorted bam files using SAMtools v.1.3.1 [46]. Assembled transcript read counts and TMM (trimmed mean of M)-normalized abundance estimates were calculated using RSEM [47] employing the Trinity helper script align_and_estimate_abundance.pl with the bowtie2 read alignments and the abundance_estimates_to_matrix.pl script. An exploratory differential expression analysis comparing the two leaf samples with a single root replicate was performed with edgeR [48] using the suggested protocol for no replicates from the edgeR manual. Comparisons of read counts were made using an exactTest with an assumed dispersion value of 0.1. GO terms were assigned to transcripts based on BLASTX hits to the SwissProt database, and enrichment analysis was performed using the Bioconductor package GOseq [49]. GO term enrichment was visualized using the R package metacoder [50]. GO term ids were converted to GO terms using WEGO [51].

Microsatellite simple sequence repeats (SSRs) were identified in assembled transcripts using the MIcroSAtellite identification tool (MISA) (http://pgrc.ipk-gatersleben.de/misa/) [52]. The minimum repeat limits were six for dinucleotides, and five for tri-, tetra-, penta-, and hexa-nucleotides. Primers for SSRs were identified using Primer3 [53] with default configuration settings.

### 2.4. Comparative Transcriptome Analysis of Anacardiaceae

The poison ivy transcriptome was checked for completeness by comparing gene content with the sequenced transcriptomes of closely related species. The assembled transcriptome for mango (*Mangifera indica* (L.)) was downloaded from NCBI (BioProject: PRJNA192676). Illumina sequencing data of transcriptomes from *Rhus chinensis* mill (SRA: SRX2011023) pistachio (*Pistacia chinensis;* SRA: SRX357356) and the Japanese lacquer tree (*Toxicodendron vernicifluum*; SRA: DRX055614) were downloaded from the NCBI SRA database and individually de novo assembled using Trinity. Predicted peptide sequences were generated from transcript nucleotide sequences using the Trinity package Transdecoder. Nucleotide transcript sequences from the five transcriptomes were then clustered using get_homologues-est v. 20170302 with options for MCL clustering “-M”, report all clusters “-t 0”, and report gene composition analysis “-c” [54]. A Venn diagram of shared gene clusters was generated using the R package systemPipeR [55]. GO term enrichment of core genes found in all analyzed taxa was determined using GOseq.

Sequenced Illumina paired reads and assembled transcripts were submitted to the NCBI SRA and TSA databases, respectively, under BioProject ID PRJNA393598.

## 3. Results and Discussion

### 3.1. De Novo Assembly and Quality Control

There are currently very few publically available sequences from *T. radicans*. As of 4 February 2017, there were 96 nucleotide sequences and 25 protein sequences in NCBI’s public databases for *T. radicans*. Most of these nucleotide sequences are gene fragments or intergenic regions used in phylogenetic population studies [56]. In order to comprehensively identify all expressed genes in *T. radicans* primary organs (leaves and roots), we sequenced the transcriptomes of two biological replicates comprised of roots or true leaves, each derived from >10 axenic poison ivy seedlings. Each of the four samples was submitted for RNA-seq sequencing on an individual lane on an Illumina HiSeq 2000 in order to maximize the sequencing depth of each sample. Three of the four samples produced ~100 million 100 bp paired reads (Table 1).

**Figure 1:**
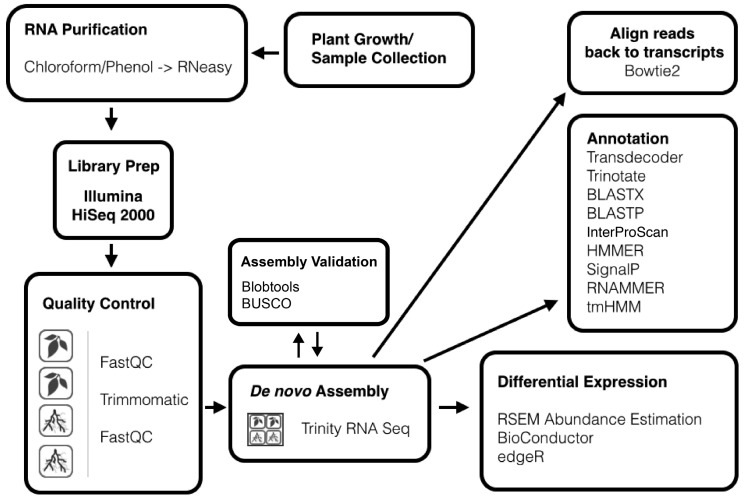
Process for de novo RNA-seq assembly and annotation. RNA from two replicates each of roots and leaves was sequenced with an Illumina HiSeq 2000. Trinity RNA seq was used to de novo assemble a reference transcriptome from the combined paired reads from all four *T. radicans* leaves and roots samples. Assembled transcripts were then annotated using a variety of programs.

De novo assembled Illumina and/or Roche 454 (Roche, Basel, Switzerland) high-throughput RNA-seq data for non-model plants and other organisms has rapidly replaced previous methods for gene discovery. Transcriptomes for safflower [57], parasitic weed *Cuscuta pentagona* [58], dogwood tree [59], *Chlorophytum borivilianum* [60], chili pepper [61], and many other non-model organisms have recently been published. Most studies used one or more of Trinity [36], Oases [62], or SOAPdenovo-Trans [63] assemblers, with comparable results [59]. Trinity was chosen for this analysis due to its excellent reputation and built-in support for downstream analyses; including the management of annotations as well as differential expression analysis. A published Trinity pipeline [40] provided a good starting reference with the addition of a few modifications (Figure 1).

The read quality of each of the four samples was checked using FastQC before and after quality trimming with Trimmomatic. Leaves replicates A and B, as well as Roots replicate B, each had ~100 million paired reads, while Roots replicate A had relatively fewer reads (~30 million). Trimmed reads from all four datasets (Roots A–B, and Leaves A–B) were combined (289,325,296 paired reads in total, ~100 bp each) and de novo assembled into an initial assembly using Trinity RNA-seq employing an adapted form of a published pipeline [40] for this analysis (Figure 1).

**Figure 2:**
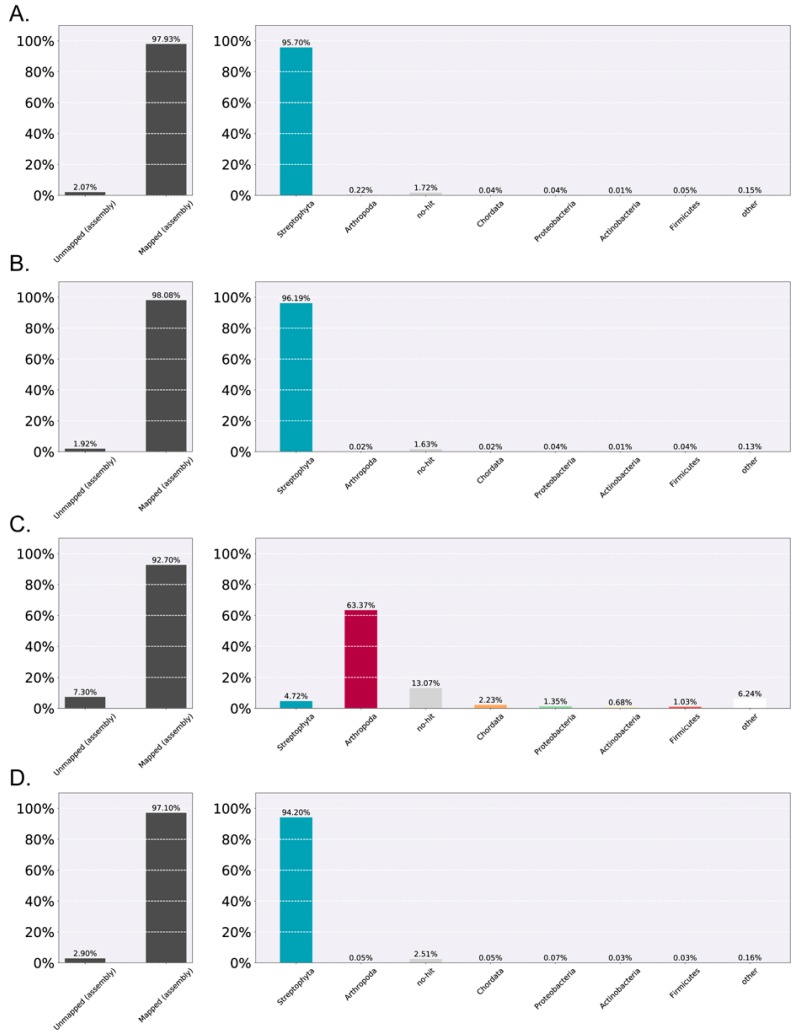
Transcript taxonomy annotation summary as determined by blobtools and BLASTX searches of the NCBI nr database. Transcript taxonomy annotation summary as determined by blobtools and BLASTX searches of the NCBI nr database for the four-sample de novo assembly. Annotated transcripts are displayed as percent of paired reads mapped to assembled transcripts of a given taxonomic grouping. (**A**) Leaves A sample summary; (**B**) Leaves B sample summary; (**C**) Roots A sample summary; (**D**) Roots B sample summary.

An unexpectedly large number of transcripts had top BLAST hits to Metazoa (particularly various insects) and Bacteria. This was surprising because the poison ivy plants were grown under sterile culture conditions. Blobplots of each sequenced dataset revealed that the poor quality Root A sample was primarily composed of arthropod and bacterial annotated transcripts and was thus the primary source of contamination (Supplementary Figures S1–S4). This contamination was found at much lower or non-existent levels in the other three samples (Figure 2).

Illumina sequencing is very sensitive and can produce reads corresponding to mRNAs at very low expression levels. Therefore, minimizing the contamination of samples becomes increasingly important, as is the correct identification of assembled transcripts stemming from contamination. It is difficult to germinate untreated *T. radicans* seedlings without fungal contamination that often results in the blighting of poison ivy seedlings [31] by *Colletotrichum fioriniae* [64], which infects both plant and insect hosts [65,66]. For this reason, axenic poison ivy seedlings were used for RNA isolation. Thus, it was expected that fungal-annotated transcripts might be abundant contaminants in the *T. radicans* transcriptomes. However, that was not the case. Instead, the predominant contaminating sequences initially seemed to be derived from metazoans.

The metazoan contamination in this dataset was largely confined to one root sample, and could have originated from a variety of possible sources. This foreign RNA (or DNA) may have been introduced during either poison ivy RNA purification or during library preparation. No metazoan samples were loaded on the same flow cell used for all the poison ivy samples, so cross contamination during flow cell loading was not likely the source of this contamination. The plants were grown in sterile-culture conditions in closed magenta boxes or phytatrays without apparent insect contamination of the visibly axenic poison ivy seedlings. A possible source of insect nucleic acid contamination could have been from the tap water used to clean the reusable polypropylene Oakridge screw capped tubes that were used during the initial plant RNA extractions, although those tubes were pre-treated with RNaseZAP (Ambion, Thermofisher Scientific, Waltham, MA, USA) to inactivate any contaminating RNase activity and were then rinsed with DEPC-treated water. Contamination may also have occurred during library preparation, as multiple samples from a variety of organisms were being simultaneously prepared for sequencing at the fee-for-service core lab facility. Whatever the source of metazoan nucleic acid contamination, the absolute levels of any given metazoan transcript were typically much lower than those of plant-annotated transcripts, as evidenced by their consistently very low coverage values in the blobplots in each of the high quality samples (Supplementary Figures S1–S4). Therefore, the proportion of metazoan contaminating sequences in the poor quality Root A sample was aberrantly large, and necessitated its exclusion from a subsequent reference de novo assembly.

**Figure 3:**
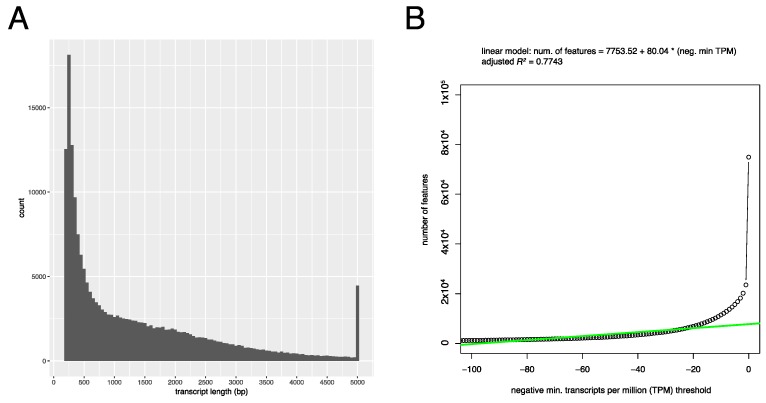
Assembly transcript length distribution and estimation of expressed transcripts. a. Assembly transcript length distribution. (**A**) Histogram shows the binned counts of the lengths of individual transcripts represented by 50 bp bins. Sequences with length greater than 5000 were binned together. The minimum transcript length reported by Trinity is 200 bp. (**B**) Estimation of expressed number of transcripts supported by the sequenced dataset. Plotted as circles are the number of transcripts with a minimum transcripts per million (TPM) value in any one sample at each threshold (plotted as negative values). A linear regression model fit to values between –100 and –10 neg. min. TPM is shown as a green line. The y-intercept (7753.52) indicates the estimated number of expressed transcripts supported by the sequenced dataset.

The trimmed reads from the three high quality samples (Leaves A–B, and Roots B) were then assembled together using Trinity to produce a high quality poison ivy reference de novo transcriptome. In total, Trinity assembled 74,937 components (a component is defined as all transcripts derived from a single de Bruijn graph) comprised of 197,641 transcript isoforms (Table 1). The average contig length was 1381 bp and the N50 was 2363 bp. When considering transcript expression, the 12,182 most highly-expressed transcripts represented 90% of the total expression data (Ex90) and had an N50 (Ex90N50) of 2077 bp. Linear regression analysis of non-normalized transcripts per million (TPM) values supported 7753 expressed components (Figure 3B). A large proportion of assembled transcripts were between 200 and 400 bases long, close to the minimum length (200 bp) of transcripts reported by Trinity (Figure 3A). Aligning paired reads to transcripts using Bowtie2 to assess overall assembly quality revealed that 94.61–95.94% of reads from each of the three high quality datasets mapped to assembled transcripts.

The presence or absence of known single-copy plant genes was used to assess the completeness of the assembled transcriptome. The BUSCO pipeline identified the presence of greater than 90% of the single-copy plant gene orthologs in the assembled poison ivy transcriptome (BUSCO code “C:90.7%(S:31.3%,D:59.4%),F:5.9%,M:3.4%,n:1440”). A large proportion of these complete genes were identified in duplicate (59.4%), suggesting that they were present and de novo assembled into multiple transcript isoforms. Just 49 (3.4%) of the 1440 tested single copy genes were missing from the de novo assembly, while 5.9% were fragmented or partially present.

### 3.2. Transcriptome Annotation

**Figure 4:**
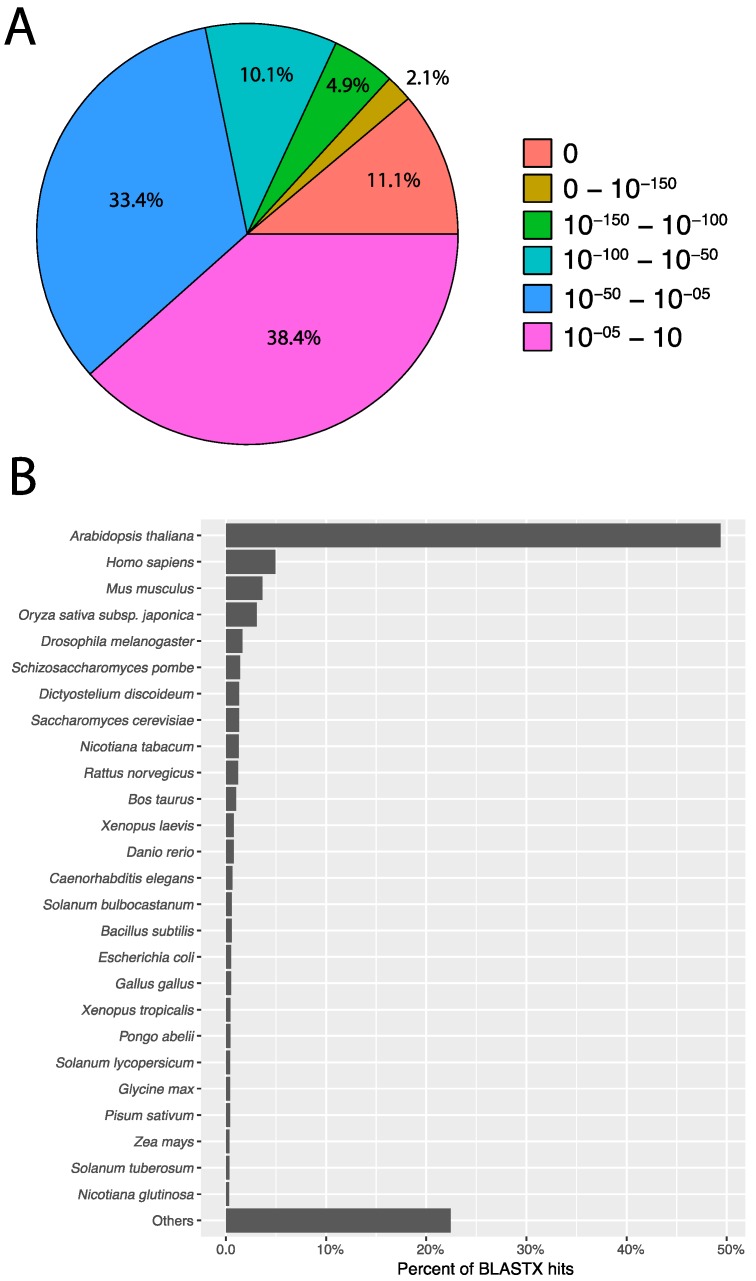
BLAST search results and species distribution. (**A**) E-value distribution for best BLASTX hit of transcripts against the SwissProt database. (**B**) Distribution of species for best BLASTX hit of transcripts against the SwissProt database.

BLASTX searches of each transcript were performed to annotate individual transcripts based on similarity to known protein sequences in the SwissProt and NCBI nr database. Of the 162,873 Trinity transcripts with BLAST hits against NCBI nr, blobtools annotated 100,918 transcripts as likely Streptophyta in origin. The rest were annotated as undefined Eukaryota (2574), Chordata (24,148), Arthropoda (3868), and a variety of bacteria, fungi, diatoms, and other microorganisms. Likewise, 79% of transcripts had a BLASTX hit against the SwissProt database (Table 1). The most common top BLAST hit to SwissProt for transcripts was to the model plant *Arabidopsis thaliana* (80,443 transcripts) (Figure 4B). Other plants had relatively large numbers of BLAST hits as would be expected for a plant transcriptome, though other non-plant organisms such as humans and mice, as well as some fungi and bacteria, had substantial numbers of BLAST hits. These are likely from contaminants, or they may be transcripts with no close homolog in other sequenced plants. E-values of BLASTX hits were generally small, with 61% of top blast hits having *e*-values less than 1e-05 and 28.2% had e-values less than 1e-50 (Figure 4A).

Some transcripts annotated as coming from a non-plant source, or not annotated at all, may also be novel plant genes not previously sequenced in other members of Viridiplantae. Therefore, their closest sequenced homologs could be from fungi or other Eukarya. Sequencing the *T. radicans* genome would reveal if these transcripts are fungal or metazoan in origin or if they are novel plant genes in poison ivy.

BLASTX hits were also used to annotate transcripts with the KEGG pathway database. KEGG annotations were assigned to 119,797 transcripts, or 60.6% of assembled transcripts. Transcripts were also annotated with a number of other annotation software tools, including signal peptide prediction with SignalP, transmembrane domain prediction with tmHMM, and ribosomal RNA prediction with RNAMMER.

### 3.3. Gene Oontology Term Analysis

**Figure 5:**
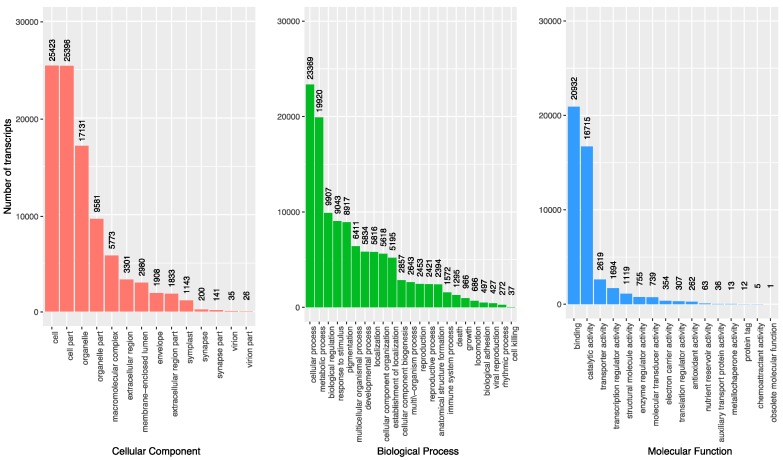
Distribution of top GO term categories. Distribution of most abundant GO terms for each of three major GO term hierarchies from GO terms assigned to *T. radicans* transcripts.

Gene Ontology (GO) terms were assigned to transcripts using the Trinotate package and SwissProt database BLASTX results. These terms represent common, broad functions and cellular components and provide a birds-eye view of the represented genes. GO terms were successfully assigned to 31,446 transcripts, or 15.9% of the assembled transcriptome. The most abundant GO terms in each of the three main GO hierarchies (cellular components, biological processes, and molecular functions) for the poison ivy transcriptome were quantified (Figure 5). The most common cellular components included cell, organelle, and organelle parts GO terms. The most abundant biological process GO terms included cellular process, metabolic process, and biological regulation, while the most abundant molecular functions included binding and catalytic activity.

### 3.4. Conserved Protein Domain Analysis with InterProScan

An InterProScan analysis was also performed in order to further annotate the assembled transcripts with protein functional domains. InterProScan annotated 128,448 transcripts, or 65% of total transcripts. The 10 most abundant InterProScan domains (Table 2) include common protein functions such as kinase domains, Leucine-rich repeat (LRR) domains, and NAD(P) binding domains. As InterProScan annotates specific protein functional domains rather than the protein as a whole, and this analysis provides additional annotation information for transcripts for which there are few or no characterized homologs.

### 3.5. Comparison of Leaf and Root Gene Expression

After removing the poor quality root sample and transcriptome re-assembly, an exploratory differential expression analysis was performed with the three high quality samples. While not statistically significant due to the single Root sample B, this analysis identified 8602 transcripts and 3363 components that are potentially differentially expressed (Benjamini-Hochberg adjusted *p*-value < 0.05, log2FC > 2) or differentially present between leaves and roots. As expected, GO term enrichment analysis of these genes revealed enrichment for chloroplast and photosynthesis associated GO terms in genes up regulated in leaves vs. roots. Oxidoreductase activity, DNA replication activity, and heme binding activity-associated GO terms, among others, were enriched in genes up regulated in roots vs. leaves (Supplementary Tables S1 and S2, Supplementary Figures S5–S7). In addition, there were 11,186 components/genes present (have at least one read mapping) in roots but not in leaves, and 16,957 components/genes present in leaves but not in roots. Taken together, these data indicated that sampling from both leaves and roots provided a broader representation of poison ivy expressed genes than either organ alone.

### 3.6. Pan-Anacardiaceae Transcriptome Comparison

**Figure 6:**
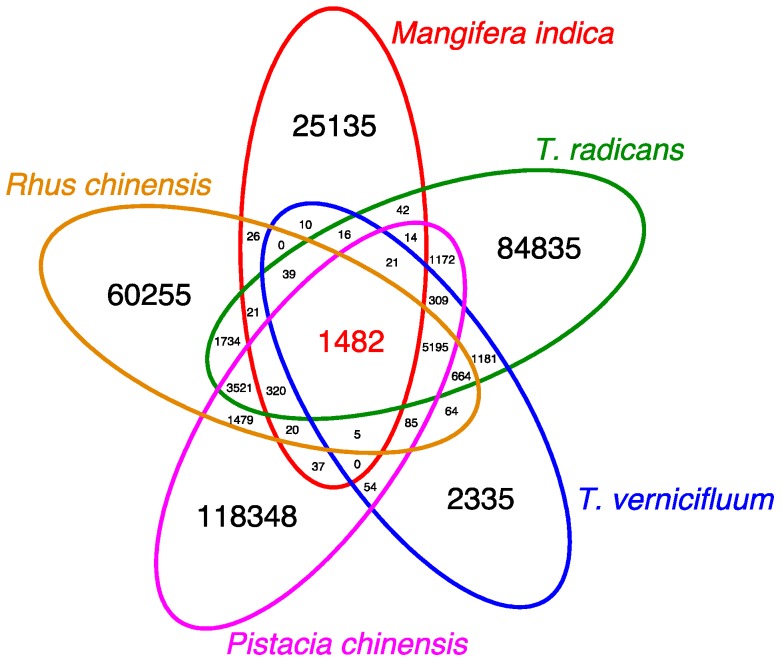
Anacardiaceae pan-transcriptome Venn diagram. Counts of orthologous transcripts shared by one or more members of Anacardiaceae are represented in each segment. The count of orthologous transcripts shared by all (core) is colored red. Regions are not to scale.

Another method to gauge transcriptome assembly quality involves clustering transcripts with those from a closely-related species. Currently, the only members of Anacardiaceae with publicly available transcriptome data are the mango tree (*Mangifera indica* (L.)), Chinese pistache tree (*Pistacia chinensis*), *Rhus chinensis*, and the Japanese lacquer tree (*Toxicodendron vernicifluum*). The Japanese lacquer tree produces urushiol, whereas mango produces related alkylresorcinols [67]. Transcriptomes for these taxa were downloaded or de novo assembled using Trinity, and compared with the *T. radicans* transcriptome. Get_homologues-est output produced 308,419 total gene clusters (individual clusters may contain more than one transcript isoform per taxa). There were 1,482 genes shared by all five species, and 7062 shared by four or more, including 5,195 genes shared by *T. radicans*, *R. chinensis*, *P. chinensis*, and *T. vernicifluum*, but not *M. indica*. The largest proportion of transcripts were those found in only one member of the five taxa and not the others (Figure 6), which is likely due to differing sequencing depth, analyzed plant tissue samples, environmental conditions, and contamination. The tissues sampled for RNA extraction differed for most of these sampled species: with *T. radicans* representing leaf and root tissues, *M. indica* and *T. vernicifluum* representing only leaf tissue, *R. chinensis* representing insect-induced gall tissue, and *P. chinensis* representing combined bud/leaf/flower/seed tissue. The 1482 genes shared by all members of Anacardiaceae were enriched in GO terms for essential cell components and cell metabolism, and plant associated GO terms (plastid, chloroplast, Supplementary Table S3).

### 3.7. SSR Identification and Primer Design

**Figure 7:**
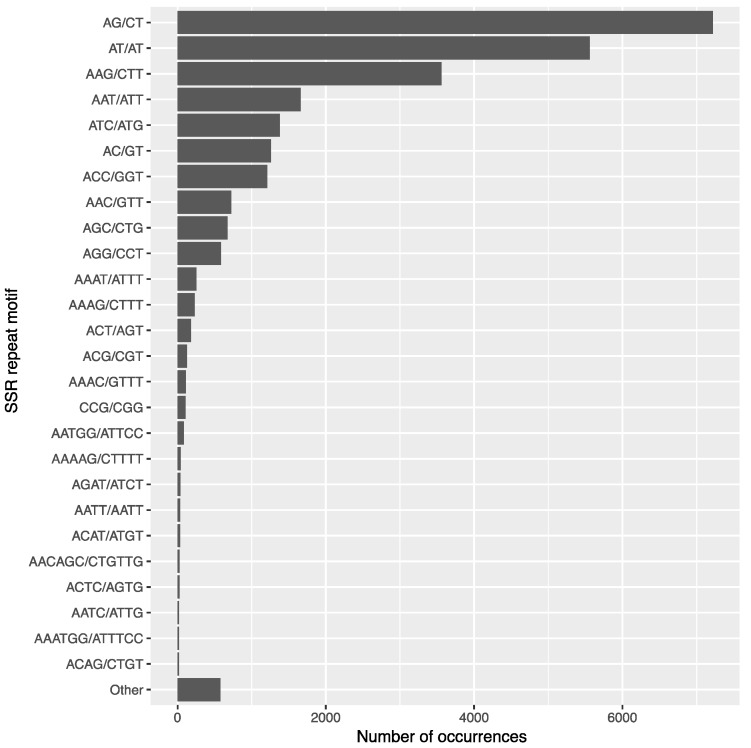
Most abundant SSR sequence motifs. Distribution of the most abundant SSR repeat motif sequences identified in assembled transcripts.

Microsatellites are simple sequence repeats (SSRs) of one to several nucleotides commonly found in the genome of Eukaryotic organisms. These repeating regions can be used to characterize differences between populations and individuals. The MIcroSAtellite identification tool (MISA) was used to screen for SSRs in the assembled transcriptome in order to build a reference of poison ivy microsatellites. When looking for repeats of two to six bases, MISA identified 25,781 SSRs in 21,075 transcripts (Table 3). A majority (94%) of repeats were di- or tri-nucleotide repeats, particularly AG/CT and AT/TA (Figure 7). The most common tri-nucleotide repeat was AAG/CCT, which was the third most abundant SSR motif. Primer3 was used to generate primer pairs targeting these SSR regions (Supplementary Table S4). The identified SSRs and primer sequences provide a resource for poison ivy population genetics and future investigations into the genetic diversity of large populations of poison ivy.

## 4. Conclusions

This work provides an initial transcriptome framework for investigating the under-studied allergenic noxious weed poison ivy in North America. The poison ivy transcriptome was sequenced in two primary vegetative tissues and de novo assembled for the first time, representing the first example of poison ivy to be sequenced en mass. The transcriptome was extensively annotated and filtered for contaminants. Given the established capacity for poison ivy to respond to anticipated future elevated atmospheric CO_2_ levels by growing faster, accumulating more biomass, and becoming more allergenic [15,16], combined with the annual 25–40 million people who are sensitized to poison ivy allergenic dermatitis [1,2], there is strong justification for molecular investigations of poison ivy ecology and physiology during the progression of the Anthropocene. To this end, the poison ivy transcriptome is a valuable resource for hypotheses-driven gene discovery of (as yet only postulated) enzyme activities involved in urushiol biosynthesis [27], and increased plant vigor in response to elevated atmospheric CO_2_ levels [15,16]. The identification of more than 23,000 SSRs greatly expands the number of polymorphic loci beyond the current 42 microsatellite markers for poison ivy [68]. Detailed studies of poison ivy population genetics would do much to clarify the genetic basis for the current *T. radicans* subspecies that are currently defined by all too variable morphological characters and biogeography [12,69]. Genetic characterization of poison ivy populations is further justified by accession-level differential biometric responses, strongly suggesting that poison ivy is differentially adapting to different local environments in North America [29].

Advances in genomic techniques have spurred novel means of understanding how aggressive and/or invasive plants grow and interact with their environment. Manipulation of weeds at the molecular level may provide future alternatives to weed control augmenting herbicide-based methods of weed control, and prompt a move towards “molecular weed science” approaches.

## Figures and Tables

**Table 1:** Paired-end read quality per sample.

Sample	Leaves A	Leaves B	Roots A	Roots B	Total
Total # of paired reads	97,494,163	93,384,797	30,270,702	128,496,278	349,645,940
Total # of paired reads after trimming	87,835,193	81,637,895	14,779,168	105,073,040	289,325,296
% of paired reads surviving trimming	90.09%	87.42%	48.82%	81.77%	
GC content	46%	46.5%	40%	47%	
Total # of assembled contigs					197,641
# of Trinity components					74,937
Total assembled bases					273,066,414
Mean contig length (bp)					1381.63
Contig N50 (bp)					2363
Contig GC%					39.05
Longest contig (bp)					16,102
# contigs > 1 kb					93,444
Contigs with SwissProt BLASTX hits					156,935
Contigs with KEGG annotation					119,797
Contigs with GO annotation					132,208

**Table 2:** Ten most abundant InterProScan domains.

Domain	Description	Transcripts with Domain
IPR027417	P-loop containing nucleoside triphosphate hydrolase	9503
IPR000719	Protein kinase domain	7405
IPR032675	Leucine-rich repeat domain, L domain-like	3946
IPR011009	Protein kinase-like domain	2937
IPR016040	NAD(P)-binding domain	2652
IPR020683	Ankyrin repeat-containing domain	2482
IPR011990	Tetratricopeptide-like helical domain	2273
IPR029058	Alpha/Beta hydrolase fold	2022
IPR000504	RNA recognition motif domain	1867
IPR012677	Nucleotide-binding alpha-beta plait domain	1866

**Table 3:** Single sequence repeats (SSR) marker results.

**MISA SSR Settings**	**Count**
Total number of sequences examined	197,641
Total size of examined sequences (bp)	273,066,414
Total number of identified SSRs	25,781
Number of SSR containing sequences	21,075
Number of sequences containing more than 1 SSR	3764
Number of SSRs present in compound formation	1843
**Unit Size**	**Number of SSRs**
di-nucleotide	14,044
tri-nucleotide	10,211
quad-nucleotide	835
tetra-nucleotide	316
hexa-nucleotide	375

## Supplementary Materials

Supplementary materials can be found at www.mdpi.com/2073-4425/8/11/317/s1. Figure S1: Blobplot of Leaf A, Figure S2: Blobplot of Leaf B, Figure S3: Blobplot of Root A, Figure S4: Blobplot of Root B, Figure S5: Relative gene expression between roots and leaves in genes matching GO term “Biological Processes”, Figure S6: Relative gene expression between roots and leaves in genes matching GO term “Cellular Component”, Figure S7: Relative gene expression between roots and leaves in genes matching GO term “Molecular Function”, Table S1: GO terms enriched in leaves compared to root tissue, Table S2: GO terms enriched in root vs. leaf tissue, Table S3: GO terms enriched in genes core to Anacardiaceae, Table S4: SSR primer sequences.

## References

[1] Epstein W.L. (1987). Plant-induced dermatitis. Ann. Emerg. Med..

[2] Epstein W.L. (1994). Occupational poison ivy and oak dermatitis. Derrmatol. Clin..

[3] Gayer K.D., Burnett J.W. (1988). Toxicodendron dermatitis. Cutis.

[4] Mitchell J., Rook A. (1979). Botanical Dermatology: Plants and Plant Products Injurious to the Skin.

[5] Hill G.A., Mattacotti V., Graha W.D. (1934). The toxic principle of the poison ivy. J. Am. Chem. Soc..

[6] Majima R. (1922). Uber den hauptbestandteil des japan-lacs, viii. Mitteilung: Stellung der doppelbindungen in der seitenkette des urushiols und beweisfuhrung, dab das urushiol eine mischung ist. Ber. Dtsch. Chem. Ges..

[7] Majima R., Cho S. (1907). Ueber einen hauptbestandteil des japanischen lackes. Ber. Dtsch. Chem. Ges..

[8] Markiewitz K.H., Dawson C.R. (1965). On the isolation of the allegenically active components of the toxic principle of poison ivy. J. Org. Chem..

[9] Symes W.F., Dawson C.R. (1953). Separation and structural determination of the olefinic components of poison ivy urushiol, cardanol and cardol. Nature.

[10] Craig J.C., Waller C.W., Billets S., Elsohly M.A. (1978). New GLC analysis of urushiol congeners in different plant parts of poison ivy, *Toxicodendron radicans*. J. Pharmacol. Sci..

[11] Aziz M., Sturtevant D., Winston J., Collakova E., Jelesko J.G., Chapman K.D. (2017). MALDI-MS imaging of urushiols in poison ivy stem. Molecules.

[12] Gillis W.T. (1971). The systematics and ecology of poison-ivy and the poison-oaks (Toxicodendron, Anacardiaceae). Rhodora.

[13] Penner R., Moodie G.E., Staniforth R.J. (1999). The dispersal of fruits and seeds of poison-ivy, *Toxicodendron radicans*, by ruffed grouse, *Bonasa umbellus*, and squirrels, *Tamiasciurus hudsonicus* and *Sciurus carolinensis*. Can. Field-Nat..

[14] Talley S.M., Lawton R.O., Setzer W.N. (1996). Host preferences of *Rhus radicans* (Anacardiaceae) in a southern deciduous harwood forest. Ecology.

[15] Mohan J.E., Ziska L.H., Schlesinger W.H., Thomas R.B., Sicher R.C., George K., Clark J.S. (2006). Biomass and toxicity responses of poison ivy (*Toxicodendron radicans*) to elevated atmospheric CO2. Proc. Natl. Acad. Sci. USA.

[16] Ziska L.H., Sicher R.C., George K., Mohan J.E. (2007). Rising atmospheric carbon dioxide and potential impacts on the growth and toxicity of poison ivy (*Toxicodendron radicans*). Weed Sci..

[17] Gillis W.T. (1975). Poison ivy and its kin. Arnoldia.

[18] Allen B.P., Pauley E.F., Sharitz R.R. (1997). Hurricane impacts on liana populations in an old-growth southeastern bottomland forest. J. Torrey Bot. Soc..

[19] Allen B.P., Sharitz R.R., Goebel P.C. (2005). Twelve years post-hurricane liana dynamics in an old-growth southeastern floodplain forest. For. Ecol. Manag..

[20] Catling P.M., Sinclair A., Cuddy D. (2002). Plant community composition and relationships of disturbed and undisturbed alvar woodland. Can. Field-Nat..

[21] Martin A.C., Zim H.S., Nelson A.L. (1951). American Wildlife & Plants.

[22] Popay I., Field R. (1996). Grazing animals as weed control agents. Weed Technol..

[23] Senchina D.S. (2008). Fungal and animal associates of *Toxicodendron* spp. (Anacardiaceae) in North America. Perspect. Plant Ecol. Evol. Syst..

[24] Suthers H.B., Bickal J.M., Rodewald P.G. (2000). Use of successional habitat and fruit resources by songbirds during autumn migration in central New Jersey. Wilson Bull..

[25] Senchina D.S. (2005). Beetle interactions with poison ivy and poison oak (*Toxicodendron* p. Mill. Sect. *Toxicodendron*, Anacardiaceae). Coleopt. Bull..

[26] Senchina D.S., Summerville K.S. (2007). Great diversity of insect floral associates may partially explain ecological success of poison ivy (*Toxicodendron radicans subps negundo greene gillis*, Anacardiaceae). Gt. Lakes Entomol..

[27] Dewick P.M. (1997). Medicinal Natural Products: A Biosynthetic Approach.

[28] Giessman T.A., Bernfeld P. (1967). The biosynthesis of phenolic plant products. The Biogenesis of Natural Compounds.

[29] Jelesko J.G., Benhase E.B., Barney J.N. (2017). Differential responses to light and nutrient availability by geographically isolated poison ivy accessions. Northeast. Nat..

[30] Johnson R.A., Baer H., Kirkpatrick C.H., Dawson C.R., Khurana R.G. (1972). Comparison of the contact allergenicity of the four pentadecylcatechols derived from poison ivy urushiol in human subjects. J. Allergy Clin. Immunol..

[31] Benhase E.B., Jelesko J.G. (2013). Germinating and culturing axenic poison ivy seedlings. HortScience.

[32] Murashige T., Skoog F. (1962). A revised medium for rapid growth and bio assays with tobacco tissue cultures. Physiol. Plant..

[33] Ausubel F.A., Brent R., Kingston R.E., Moore D.D., Seidman J.G., Smith J.A., Struhl K. (2006). Current Protocols in Molecular Biology.

[34] Andrews S. FastQC: A Quality Control Tool for High Throughput Sequence Data. http://www.Bioinformatics.Babraham.Ac.Uk/projects/fastqc.

[35] Bolger A., Lohse M., Usadel B. (2014). Trimmomatic: A flexible trimmer for illumina sequence data. Bioinformatics.

[36] Grabherr M., Haas B., Yassour M., Levin J., Thompson D., Amit I., Adiconis X., Fan L., Raychowdhury R., Zeng Q. (2011). Full-length transcriptome assembly from RNA-Seq data without a reference genome. Nat. Biotechnol..

[37] Langmead B., Salzberg S. (2012). Fast gapped-read alignment with Bowtie 2. Nat. Methods.

[38] Kumar S., Jones M., Koutsovoulos G., Clarke M., Blaxter M. (2013). Blobology: Exploring raw genome data for contaminants, symbionts and parasites using taxon-annotated GC-coverage plots. Front. Genet..

[39] Simao F., Waterhouse R., Ioannidis P., Kriventseva E., Zdobnov E. (2015). BUSCO: Assessing genome assembly and annotation completeness with single-copy orthologs. Bioinformatics.

[40] Haas B., Papanicolaou A., Yassour M., Grabherr M., Blood P., Bowden J., Couger M., Eccles D., Li B., Lieber M. (2013). De novo transcript sequence reconstruction from RNA-Seq using the Trinity platform for reference generation and analysis. Nat. Protocols.

[41] Finn R., Clements J., Eddy S. (2011). Hmmer web server: Interactive sequence similarity searching. Nucleic Acids Res..

[42] Finn R., Bateman A., Clements J., Coggill P., Eberhardt R., Eddy S., Heger A., Hetherington K., Holm L., Mistry J. (2014). Pfam: The protein families database. Nucleic Acids Res..

[43] Petersen T., Brunak S., von H.G., Nielsen H. (2011). Signalp 4.0: Discriminating signal peptides from transmembrane regions. Nat. Methods.

[44] Krogh A., Larsson B., von H.G., Sonnhammer E. (2001). Predicting transmembrane protein topology with a hidden Markov model: Application to complete genomes. J. Mol. Biol..

[45] Lagesen K., Hallin P., Rodland E., Staerfeldt H., Rognes T., Ussery D. (2007). Rnammer: Consistent and rapid annotation of ribosomalRNA genes. Nucleic Acids Res..

[46] Li H., Handsaker B., Wysoker A., Fennell T., Ruan J., Homer N., Marth G., Abecasis G., Durbin R., 1000 Genome Project Data Processing Subgroup (2009). The sequence alignment/map format and SAMtools. Bioinformatics.

[47] Li B., Dewey C. (2011). RSEM: Accurate transcript quantification from RNA-Seq data with or without a reference genome. BMC Bioinform..

[48] Robinson M., McCarthy D., Smyth G. (2010). edgeR: A bioconductor package for differential expression analysis of digital gene expression data. Bioinformatics.

[49] Young M., Wakefield M., Smyth G., Oshlack A. (2010). Gene ontology analysis for RNA-seq: Accounting for selection bias. Genome Biol..

[50] Foster Z., Sharpton T., Grunwald N. (2017). Metacoder: An R package for visualization and manipulation of community taxonomic diversity data. PLoS Comp. Biol..

[51] Ye J., Fang L., Zheng H.K., Zhang Y., Chen J., Zhang Z.J., Wang J., Li S.T., Li R.Q., Bolund L. (2006). WEGO: A web tool for plotting GO annotations. Nucleic Acids Res..

[52] Thiel T. Misa—Microsatellite identification tool. http://pgrc.ipk-gatersleben.de/misa/.

[53] Untergasser A., Cutcutache I., Koressaar T., Ye J., Faircloth B.C., Remm M., Rozen S.G. (2012). Primer3--new capabilities and interfaces. Nucleic Acids Res..

[54] Contreras-Moreira B., Cantalapiedra C., Garcia-Pereira M., Gordon S., Vogel J., Igartua E., Casas A., Vinuesa P. (2017). Analysis of plant pan-genomes and transcriptomes with GET_HOMOLOGUES-EST, a clustering solution for sequences of the same species. Front. Plant Sci..

[55] Backman T.W.H., Girke T. (2016). SystemPipeR: NGS workflow and report generation environment. BMC Bioinform..

[56] Nie Z.-L., Sun H., Meng Y., Wen J. (2009). Phylogenetic analysis of *Toxicodendron* (Anacardiaceae) and its biogeographic implications on the evolution of north temperate and tropical intercontinental disjunctions. J. Syst. Evol..

[57] Lulin H., Xiao Y., Pei S., Wen T., Shangqin H. (2012). The first Illumina-based de novo transcriptome sequencing and analysis of safflower flowers. PLoS ONE.

[58] Ranjan A., Ichihashi Y., Farhi M., Zumstein K., Townsley B., David-Schwartz R., Sinha N. (2014). De novo assembly and characterization of the transcriptome of the parasitic weed dodder identifies genes associated with plant parasitism. Plant Physiol..

[59] Zhang J., Ruhlman T., Mower J., Jansen R. (2013). Comparative analyses of two Geraniaceae transcriptomes using next-generation sequencing. BMC Plant Biol..

[60] Kalra S., Puniya B., Kulshreshtha D., Kumar S., Kaur J., Ramachandran S., Singh K. (2013). De novo transcriptome sequencing reveals important molecular networks and metabolic pathways of the plant, *Chlorophytum borivilianum*. PLoS ONE.

[61] Liu S., Li W., Wu Y., Chen C., Lei J. (2013). De novo transcriptome assembly in chili pepper (*Capsicum frutescens*) to identify genes involved in the biosynthesis of capsaicinoids. PLoS ONE.

[62] Schulz M., Zerbino D., Vingron M., Birney E. (2012). Oases: Robust de novoRNA-Seq assembly across the dynamic range of expression levels. Bioinformatics.

[63] Xie Y., Wu G., Tang J., Luo R., Patterson J., Liu S., Huang W., He G., Gu S., Li S. (2014). SOAPdenovo-Trans: De novo transcriptome assembly with short RNA-Seq reads. Bioinformatics.

[64] Kasson M.T., Pollok J.R., Benhase E.B., Jelesko J.G. (2014). First report of seedling blight of eastern poison ivy (*Toxicodendron radicans*) by *Colletotrichum fioriniae* in Virginia. Plant Dis..

[65] Marcelino J., Giordano R., Gouli S., Gouli V., Parker B.L., Skinner M., TeBeest D., Cesnik R. (2008). *Colletotrichum acutatum* var. Fioriniae (teleomorph: *Glomerella acutata* var. Fioriniae var. Nov.) infection of a scale insect. Mycologia.

[66] Marcelino J.A., Gouli S., Parker B.L., Skinner M., Giordano R. (2009). Entomopathogenic activity of a variety of the fungus, *Colletotrichum acutatum*, recovered from the elongate hemlock scale, *Fiorinia externa*. J. Insect Sci..

[67] Aguilar-Ortigoza C.J., Sosa V., Aguilar-Ortigoza M. (2003). Toxic phenols in various Anacardiaceae species. Econ. Bot..

[68] Hsu T.W., Shih H.C., Ku C.C., Chiang T.Y., Chiang Y.C. (2013). Characterization of 42 microsatellite markers from poison ivy, *Toxicodendron radicans* (Anacardiaceae). Int. J. Mol. Sci..

[69] Barkley F.A. (1937). A monographic study of Rhus and its immediate allies in North and Central America, including the West Indies. Ann. MO. Bot. Gard..

